# Quantitative secretome and glycome of primary human adipocytes during insulin resistance

**DOI:** 10.1186/1559-0275-11-20

**Published:** 2014-05-12

**Authors:** Jae-Min Lim, Edith E Wollaston-Hayden, Chin Fen Teo, Dorothy Hausman, Lance Wells

**Affiliations:** 1 Complex Carbohydrate Research Center, The University of Georgia, 315 Riverbend Road, 30602-4712 Athens, Georgia; 2 Department of Chemistry, The University of Georgia, 30602 Athens, Georgia; 3 Department of Biochemistry and Molecular Biology, The University of Georgia, 30602 Athens, Georgia; 4 Department of Foods and Nutrition, The University of Georgia, 30602 Athens, Georgia; 5 Department of Chemistry, Changwon National University, Changwon, Gyeongnam 641-773, South Korea

**Keywords:** O-GlcNAc, Insulin resistance, Type 2 diabetes, Adipocytokine, Tandem mass spectrometry, Shotgun proteomics, Glycomics, N-linked, O-linked

## Abstract

Adipose tissue is both an energy storage depot and an endocrine organ. The impaired regulation of the secreted proteins of adipose tissue, known as adipocytokines, observed during obesity contributes to the onset of whole-body insulin resistance and the pathobiology of type 2 diabetes mellitus (T2DM). In addition, the global elevation of the intracellular glycosylation of proteins by O-linked β-N-acetylglucosamine (O-GlcNAc) via either genetic or pharmacological methods is sufficient to induce insulin resistance in both cultured cells and animal models. The elevation of global O-GlcNAc levels is associated with the altered expression of many adipocytokines. We have previously characterized the rodent adipocyte secretome during insulin sensitive and insulin resistant conditions. Here, we characterize and quantify the secretome and glycome of primary human adipocytes during insulin responsive and insulin resistant conditions generated by the classical method of hyperglycemia and hyperinsulinemia or by the pharmacological manipulation of O-GlcNAc levels. Using a proteomic approach, we identify 190 secreted proteins and report a total of 20 up-regulated and 6 down-regulated proteins that are detected in both insulin resistant conditions. Moreover, we apply glycomic techniques to examine (1) the sites of N-glycosylation on secreted proteins, (2) the structures of complex N- and O-glycans, and (3) the relative abundance of complex N- and O-glycans structures in insulin responsive and insulin resistant conditions. We identify 91 N-glycosylation sites derived from 51 secreted proteins, as well as 155 and 29 released N- and O-glycans respectively. We go on to quantify many of the N- and O-glycan structures between insulin responsive and insulin resistance conditions demonstrating no significant changes in complex glycosylation in the time frame for the induction of insulin resistance. Thus, our data support that the O-GlcNAc modification is involved in the regulation of adipocytokine secretion upon the induction of insulin resistance in human adipocytes.

## Introduction

Type 2 diabetes mellitus (T2DM) is a rapidly growing problem in the industrialized world. In the United States, it is estimated that 8.3% of the population has diabetes [1]. T2DM results from a combination of insulin resistance and pancreatic beta-cell dysfunction [2]. The development of T2DM depends on both genetic and environmental risk factors [3]. The major environmental risk factor for the development of insulin resistance and T2DM is obesity. Increased white adipose tissue (WAT) mass during obesity causes a variety of problems related to the development of insulin resistance. It is now established that WAT acts as a lipid metabolism modulator as well as an endocrine organ [4].

The secreted proteins of adipose tissue, known as adipocytokines, can act in a paracrine or endocrine manner to modulate a variety of processes such as inflammation and whole-body energy homeostasis. During obesity, adipocytokine secretion is altered, which can lead to the development of whole-body insulin resistance and T2DM [5,6]. Although many adipocytokines have been identified using methods such as mass spectrometry-based proteomics, the alteration in the adipose tissue secretome for normal vs. insulin resistant human adipose tissue has not been well-defined [7]. Several adipocytokines identified in rodents have different expression patterns in humans highlighting the need for quantitative proteomic studies in human adipose tissue [8,9]. To achieve a more physiological representation of the adipose tissue secretome, we use primary human pre-adipocytes derived from human adipose tissue rather than an adipocyte cell line [10].

The regulation of adipocytokine expression during insulin resistance is still unclear in many cases. One possible way for cells to sense nutrient abundance and modulate insulin sensitivity and adipocytokine expression is through the hexosamine biosynthetic pathway (HBP). Excessive flux through the HBP results in insulin resistance, thereby limiting the amount of glucose that enters the cell and the resulting toxicity [11,12]. Flux through the HBP also modulates the expression of several adipocytokines [13-16]. The final product of the HBP is UDP-GlcNAc, which is the sugar donor for the enzyme O-GlcNAc transferase (OGT) that adds the O-linked *β*-N-acetylglucosamine (O-GlcNAc) modification onto the serine and threonine residues of nucleocytosolic proteins [17,18]. It has been shown in multiple systems that the elevation of O-GlcNAc levels is sufficient to cause insulin resistance [19-24]. Furthermore, the overexpression of OGT in the peripheral tissues of mice results in both glucose disposal defects and hyperleptinemia providing further evidence that the O-GlcNAc modification causes insulin resistance and the dysregulation of adipocytokine expression [21]. We have previously characterized and quantified the secretome of rodent adipocytes during insulin resistance generated by either directly or indirectly modulating O-GlcNAc levels [25]. We use a similar approach in this study. Insulin resistance is induced in primary human adipocytes by either the classical method of chronic hyperinsulinemia and hyperglycemia or by directly elevating O-GlcNAc levels using O-GlcNAcase (OGA) pharmacological inhibitors. To identify and quantify the secretome we use a proteomic approach with reverse phase (RP) liquid chromatography-nanospray-tandem mass spectrometry (LC-NS-MS/MS). We identify a total of 190 secreted proteins from primary human adipocytes. We compare the relative abundance of the adipocytokines during insulin responsive versus insulin resistant conditions using spectral counts. We report that 20 proteins are upregulated and 4 are downregulated when primary human adipocytes are shifted from insulin sensitive to insulin resistant conditions.

Glycosylation is one of the most common post-translational modifications (PTMs) of proteins, especially of secreted proteins [26-34]. In certain disease states, the glycome and the enzymes responsible for the glycan complexity can be altered [35,36]. It has been suggested that the increased pool of UDP-GlcNAc during excessive nutrient flux alters glycosyltransferase activity and the degree of N-glycan branching [37]; however, another study reported no change in complex glycosylation during elevated HBP flux [38]. Determining the glycome in different disease states is important for the future identification of structure-function relationships and to discover targets for diagnostic biomarkers [25,39,40]. The glycome of human adipose tissue has not been previously described. Glycan analysis is challenging due to the diversity of possible structures and their low abundance [35,41-49]. We use several strategies to identify, site-map, and quantify glycans from adipose tissue using mass spectrometry (MS). We characterize a total of 155 N-linked glycans and 29 O-linked glycans using MS/MS spectra by total ion mapping (TIM) scan. Predominant N-linked and O-linked glycans can be quantified by ^13^C isotopic labeling by permethylation using heavy or light iodomethane (^13^CH_3_I and ^12^CH_3_I) and isobaric pairs of iodomethane (^13^CH_3_I or ^12^CH_2_DI) (48, 49). A total of 48 N-linked glycans and 12 O-linked glycans are compared between the insulin responsive condition and both insulin resistance conditions by calculating the ^13^C/^12^C ratios from the sum of peak areas and from non-labeled prevalence rates. We also identify 91 N-glycosylation sites derived from 51 secreted proteins using the PNGase F digestion in ^18^O water using a parent mass list method.

Herein, we describe the secretome and glycome of primary human adipocytes during insulin sensitive and insulin resistant condition. Given the important physiological roles of adipocytokines in maintaining whole-body energy homeostasis, the characterization of the primary human adipocyte secretome during insulin resistance could provide novel markers and/or therapeutic targets for the onset of insulin resistance.

## Experimental procedures

### Tissue culture and conditioned cell treatments

Cryopreserved human subcutaneous preadipocytes (number of donors: 6–7, gender of donors: female, average age: 39, and average BMI: 27.32) were purchased from Zen-Bio, Inc. (Research Triangle Park, NC). The adipose tissue culture protocols of the maintenance and differentiation from preadipocytes to adipocytes were based on Zen-Bio instruction manual (ZBM0001.01). Briefly approximately 6.7 × 10^5^ cells were cultured in a T-75 cm^2^ culture flask using preadipocyte medium (PM-1, Zen-Bio, Inc.), under a humidified atmosphere containing 5% CO_2_ in air at 37°C until they were 85-90% confluent, and then were trypsinized for 5 minutes at 37°C. After neutralization and centrifugation steps, the cell pellet was resuspended in PM-1 and seeded at an average density of 2.67 × 10^4^ cells/cm^2^ in 10-cm cell culture dishes for differentiation. 2 days after the cells reached confluence (referred to as day 0), the medium was replaced with adipocyte differentiation medium (DM-2, Zen-Bio, Inc.). On day 7, the DM-2 was removed and the cells were maintained in adipocyte maintenance medium (AM-1, Zen-Bio, Inc.). The medium was changed every 3 days. On day 15, at which the majority of the cells contained large lipid droplets, the AM-1 medium was replaced with low glucose DMEM (Cellgro, Mediatech, Inc.) containing 10% FBS (GIBCO, Invitrogen) and antibiotics (100 units/mL penicillin and 100 μg/mL streptomycin (P/S), Cellgro, Mediatech, Inc.). The medium was changed every 2 days until treatments were applied. On day 20, adipocytes were treated with different conditions, either (1) low glucose (LG) (LG, DMEM containing 10% FBS and P/S), (2) insulin (100 nM, human, Roche) in high glucose (HG + INS) (HG, DMEM containing 10% FBS and P/S), (3) PUGNAc (100 μM, TRC, Inc.) in low glucose (LG + PUGNAc) (LG, DMEM containing 10% FBS and P/S), or (4) GlcNAcstatin (20 nM, kind gift from Dr. Daan van Aalten) in low glucose (LG + PUGNAc) (LG, DMEM containing 10% FBS and P/S). After the first 24 h of incubation, the cells were washed five times (for mass spectrometry analysis) or 3 times (for immunoblotting) with low or high glucose serum-free DMEM without antibiotics and incubated for 15 min during the last rinse. After the final wash, the treatment conditions were added as above except no serum was added and the HG + INS treatment was changed to 1nM Insulin. The cells and media were harvested after 16 h of incubation.

### Secreted protein sample preparation

The conditioned media was harvested with extreme care not to disrupt underlying cells and then centrifuged once at 1800 rpm, at 4°C for 7 min. The supernatant was filtered using 1 μm syringe filters (PALL). The samples were then centrifuged again at 30000 × *g*, at 4°C for 30 min. The samples were then transferred to equilibrated spin columns (Centriprep YM-3, Amicon, Millipore) and buffer-exchanged at 2800 × *g*, at 4°C into 40 mM ammonium bicarbonate (NH_4_HCO_3_) in the presence of 1 mM dithiothreitol (DTT, Fisher Scientific) and concentrated. The samples were either quantified and prepared for immunoblotting or were denatured with 1 M urea (Sigma), reduced with 10 mM DTT for 1 h at 56°C, carboxyamidomethylated with 55 mM iodoacetamide (ICH_2_CONH_2_, Sigma) in the dark for 45 min, and then digested with 4 μg of trypsin (Promega) in 40 mM NH_4_HCO_3_ overnight at 37°C. After digestion, the peptides were acidified with 200 μL of 1% trifluoroacetic acid (TFA). Desalting was subsequently performed with C18 spin columns (Vydac Silica C18, The Nest Group, Inc.) and the resulting peptides were dried down in a Speed Vac and stored at −20°C until analysis. For the subset of samples to be analyzed for N-linked glycosylation, peptides were resuspended in 19 μL of ^18^O water (H_2_^18^O, 95%, Cambridge Isotope Laboratories, Inc.) and 1 μL of N-Glycosidase F (PNGase F, Prozyme) and allowed to incubate for 18 h at 37°C. Peptides were dried back down and resuspended in 50 μL of 40 mM NH_4_HCO_3_, with 1 μg of trypsin for 4 hr, to remove any possible C-terminal incorporation of ^18^O from residual trypsin activity, and then dried down and stored at −20°C until analysis.

### Whole cell extracts and western blots

After culture medium was removed for secreted proteins analysis, the cell monolayer was washed twice with 10 ml of ice-cold PBS and scraped in the presence of 1 ml PBS plus protease inhibitors. After removing PBS by centrifugation at 6,000 × *g* for 5 min at 4°C, the pellet was snap frozen and stored at −80°C. To prepare lysate for immunoblotting, the pellets were lysed in 20 mM Tris pH 7.5, 150 mM NaCl, 1 mM EDTA, 1% NP-40, 1:100 protease inhibitor cocktail set V, EDTA-free (Calbiochem), and 1 uM PUGNAc. Protein concentration was determined using the Pierce BCA Protein Assay Kit (Thermo Scientific) and samples were boiled in Laemli sample buffer. The immunoblots were performed essentially as described [50] using CTD 110.6 (for O-GlcNAc modified proteins) and ERK-2 (as a positive control for loading) antibodies. For secreted protein immunoblotting, the media concentration was quantified using the Bradford method and verified by Coomassie staining. Equal amounts of protein were separated by SDS-PAGE with Tris–HCl precast minigels (Bio-Rad) and transferred to nitrocellulose membranes for Western blot analysis. After blocking for at least 1 hour, membranes were incubated with the appropriate primary antibody, anti-SPARC (Abcam) or anti-Chitinase-3-like protein 1 (R&D Systems) overnight at 4°C. Membranes were incubated with the appropriate horseradish peroxidase-coupled secondary antibodies for 1 hour, followed by extensive washing and Pierce ECL detection.

### Protein extracts for glycan analysis

After the conditioned media was collected, the adipocytes were immediately washed twice with ice-cold PBS and harvested by scraping for glycan analysis. The cells were centrifuged at 12,000 × *g*, at 4°C for 15 min to remove the supernatant and debris, then snap frozen and stored at −80°C until analysis. After the cell pellets thawed on ice, the samples were subjected to Dounce homogenization in ice-cold 100% methanol. The homogenized samples were delipidated by two solvent extractions using a mixture of chloroform/methanol/water (4:8:3, v/v/v) and rocking for 3 h at room temperature as described previously [35,40]. The emulsion was centrifuged at 2800 × *g* for 15 min at 4°C to remove the supernatant. The pellets were resuspended in an acetone/water (10:1, v/v) mixture and incubated on ice for 15 min for washing. The protein pellets were collected by centrifugation and dried on a heating module at 45°C under a mild nitrogen stream (Reacti-Therm™ and Reacti-Vap™, Pierce). The dried protein powder was weighed and stored at −20°C until analysis.

### Preparation of N-linked glycans

Three mg of the protein powder was resuspended in 200 μL of 40 mM NH_4_HCO_3_ by sonication followed by boiling at 100°C for 5 min. After cooling to room temperature, 25 μL of trypsin (2 mg/mL in 40 mM NH_4_HCO_3_, Sigma) and chymotrypsin (2 mg/mL in 40 mM NH_4_HCO_3_, Sigma) were added. The samples were denatured with 250 μL of 2 M urea in 40 mM NH_4_HCO_3_, leading to a final concentration of 1 M urea, and incubated overnight (18 h) at 37°C. After digestion, the peptide samples were centrifuged and 10 μL of the supernatant was collected for protein quantification. The peptide concentrations were measured using a Pierce BCA Protein Assay Kit (Thermo Scientific). The samples were boiled at 100°C for 5 min and then acidified by adding 500 μL of 10% acetic acid (AcOH) to deactivate proteases. The samples were loaded onto an equilibrated C18 extraction column (BakerBond™, J.T.Baker), washed with 1 mL of 5% AcOH three times, and then eluted stepwise using 1 mL of 20% isopropanol in 5% AcOH, 40% isopropanol in 5% AcOH, and 100% isopropanol. The resulting glycopeptides were dried in a Speed Vac, resuspended in 48 μL 1× PNGase F reaction buffer and 2 μL PNGase F and incubated for 18 h at 37°C. Following PNGase F digestion, released oligosaccharides were separated by the C18 extraction column. The mixture was reconstituted in 5% AcOH and loaded onto an equilibrated C18 extraction column. The N-linked oligosaccharides were eluted using 1 mL of 5% AcOH three times and then collected and dried using a Speed Vac for subsequent permethylation.

### Preparation of O-linked glycans

O-linked oligosaccharides were released by reductive *β*-elimination then purified by a cation exchange resin. Three mg of delipidated protein powder was weighed and transferred into a clean glass tube. 500 μL of 50 mM sodium hydroxide (NaOH) and 500 μL of alkaline borohydride solution (a mixture of 2 M sodium borohydride (NaBH_4_, Sigma-Aldrich) in 50 mM NaOH leading to a final concentration of 1 M NaBH_4_) were added to the tube. The mixture was incubated for 16 h at 45°C on the heating block and the reaction was stopped by the addition of 10% AcOH with vortexing. The acidified mixture was loaded onto an equilibrated cation exchange resin cartridge (AG 50 W-X8, Bio-Rad) with 5% AcOH. O-linked glycans were eluted with 6 mL of 5% AcOH and then dried in a Speed Vac. The sample was resuspended in 1 mL methanol/glacial acetic acid (9:1, v/v) solution and dried on the heating module at 45°C under a mild nitrogen stream to remove borates for subsequent permethylation.

### Permethylation of glycans

To facilitate analysis of oligosaccharides by mass spectrometry, the released oligosaccharide mixtures were permethylated as described previously [35,39,51]. Briefly, glycans were resuspended in 200 μL of anhydrous dimethyl sulfoxide (DMSO, Sigma-Aldrich) and 250 μL of fresh dehydrated NaOH/DMSO reagent (mixture of 50 mg NaOH in 2 mL of anhydrous DMSO). After sonication and vortexing under nitrogen gas, 100 μL of ^12^C or ^13^C-iodomethane (^12^CH_3_I and ^13^CH_3_I, 99% of ^13^C, Sigma-Aldrich) was added and the mixtures were vortexed vigorously for 5 min. 2 mL of distilled water was added to the samples and the excess iodomethane was removed by bubbling with a nitrogen stream. Two mL of dichloromethane (CH_2_Cl_2_, Sigma-Aldrich) was added. After vigorous mixing and phase separation by centrifugation, the upper aqueous layer was removed and discarded. The nonpolar organic phase was then extracted 4 times with distilled water. Dichloromethane was evaporated on the heating module at 45°C with a mild nitrogen stream. The permethylated glycans were dissolved in adjusted volumes (15–30 μL) of 100% methanol according to the results of the protein assay. The ^12^C and ^13^C-labeled permethylated glycans were mixed in the same proportion for each experimental condition before analysis.

### Analysis using mass spectrometry

For proteome analysis of secreted proteins by liquid chromatography tandem mass spectrometry (LC-MS/MS), the peptides were resuspended with 19.5 μL of mobile phase A (0.1% formic acid, FA, in water) and 0.5 μL of mobile phase B (80% acetonitrile, ACN, and 0.1% formic acid in water) and filtered with 0.2 μm filters (Nanosep, PALL). The samples were loaded off-line onto a nanospray tapered capillary column/emitter (360 × 75 × 15 μm, PicoFrit®, New Objective) that was self-packed with C18 reverse phase (RP) resin (8.5 cm, Waters) in a nitrogen pressure bomb for 10 min at 1000 psi (~5 μL load). The samples were then separated via a 160 min linear gradient of increasing mobile phase B at a flow rate of ~200 nL/min directly into the mass spectrometer. One-dimensional LC-MS/MS analysis was performed using a linear ion trap and an Orbitrap mass spectrometer (LTQ and LTQ Orbitrap XL, Thermo Fisher Scientific Inc., San Jose, CA) equipped with a nanoelectrospray ion source at 2.0 kV capillary voltage and 200°C capillary temperature. For secretome analysis, a full ITMS (Ion trap mass spectrometry) spectrum in positive ion and profile mode was collected at 300–2000 m/z followed by 8 MS/MS events on the 8 most intense peaks with enabled dynamic exclusion using a repeat count of 2, a maximum exclusion list size of 100, and an exclusion duration of 30 s. Each MS/MS scan event was followed by CID (34% normalized collision energy), 0.25 activation Q, and 30.0 ms activation time. For N-liked glycosylation site mapping, a full FTMS (Fourier transform mass spectrometry) spectrum, typically recorded at 60000 resolution in positive ion and profile mode, was acquired at 300–2000 m/z followed by 5 data dependent MS/MS spectra of ITMS on the most intense ion peaks from parent mass list following CID (36% normalized collision energy), 0.25 activation Q, and 30.0 ms activation time. Dynamic exclusion was set at a repeat count of 2, a maximum exclusion list of 100, and an exclusion duration of 60 s.

For glycome analysis by direct infusion nanospray MS, permethylated glycans were dissolved by combining 15 μL of the isotopically mixed sample in 100% methanol plus 35 μL 1 mM NaOH in 50% methanol. They were infused directly into a linear ion trap and an Orbitrap mass spectrometer using a nanospray ion source with a fused-silica emitter (360 × 75 × 30 μm, SilicaTip™, New Objective) at 2.0 kV capillary voltage, 200°C capillary temperature, and a syringe flow rate of 0.4 μL/min. The full ITMS spectra and FTMS spectra, typically recorded at 60000 resolution in positive ion and profile mode, were collected at 400–2000 m/z for 30 s with 5 microscans and 150 maximum injection times (ms). The centroid MS/MS spectra following collision-induced dissociation (CID) were obtained from 400 to 2000 m/z at 34% and 28% normalized collision energy for N- and O-linked glycans, respectively, 0.25 activation Q, and 30.0 ms activation time by total ion mapping (TIM). Parent mass step size and isolation width were set at 2.0 m/z and 2.8 m/z, respectively, for automated MS/MS spectra with TIM scans.

### Data analysis

The resulting proteomic data was searched against a target nonredundant human (*Homo sapiens*, 5-2-07) database including the common contaminants database obtained from the human International Protein Index (IPI) protein sequence database (European Bioinformatics Institute, ftp://ftp.ebi.ac.uk/pub/databases/IPI) using the TurboSequest algorithm (BioWorks 3.3.1 SP1, Thermo Fisher Scientific Inc.) [52,53]. It was also searched against a decoy human database to determine statistically relevant peptide identification. DTA files were generated for spectra with a threshold of 15 ions and a TIC of 2e^3^ over a range of [MH]^+^ = 600–4000 for only IT data. The SEQUEST parameters were set to allow 2.0 Da (20 ppm for FT) of precursor ion mass tolerance and 0.5 Da of fragment ion tolerance with monoisotopic mass. Only strict tryptic peptides were allowed with up to two missed internal cleavage sites. Dynamic mass increases of 15.99 and 57.02 Da (15.9949 and 57.0215 Da for FT) were allowed for oxidized methionine and alkylated cysteine, respectively. In the cases where sites of N-linked glycosylation were investigated with PNGase F and ^18^O water, a dynamic mass increase of 3.0 Da (2.9883 Da for FT) was allowed for Asn residues [54]. The masses were selected between 300–2000 m/z at each charge state and 7352 total masses were obtained for parent mass list. Each sample was analyzed by four LC-MS/MS runs with different parent mass list up to 2000 because of maximum number of parent masses for FTMS and mass complexity. The results from shotgun method and parent mass list method were combined and filtered at ≥ 0.60 Final Score (Sf). For relative protein quantification, the identified peptides and proteins were statistically validated between the target and decoy database search results, and protein ratios were determined by normalized spectral counts using ProteoIQ 1.1 (Premiere BioSoft). Proteins identified by two peptides were only considered to be statistically significant at less than 1% protein false discovery rate (FDR) using the ProValT algorithm as implemented in ProteoIQ [55,56]. From the results, the subcellular location of secreted proteins was manually determined for each protein from the Human Protein Reference Database (http://www.hprd.org/), Information Hyperlinked Over Proteins (iHOP, http://www.ihop-net.org/UniPub/iHOP/) and the UniProtKB/Swiss-Prot (http://www.ebi.ac.uk/uniprot) databases. The functional categories of secretome were annotated by the Ingenuity Pathways Analysis program (IPA, Ingenuity Systems, Inc.).

The N- and O-linked glycans released from the protein power of adipocytes were analyzed by full MS following permethylation and subsequent MS/MS fragmentations for specific glycan structures. We used GlycoWorkbench (http://glycomics.ccrc.uga.edu/eurocarb/gwb/home.action) to facilitate manually interpretation of the glycan structures from the MS/MS spectra by TIM scan. The areas of each isotopic peak were calculated by their Gaussian distribution and then averaged to compare the relative abundance of each glycan between different samples. The ratio of peak areas from isotopic permethylation defined the relative abundance of each glycan.

## Results

### Insulin resistance and global O-GlcNAc levels in primary human adipocytes

**Figure 1:**
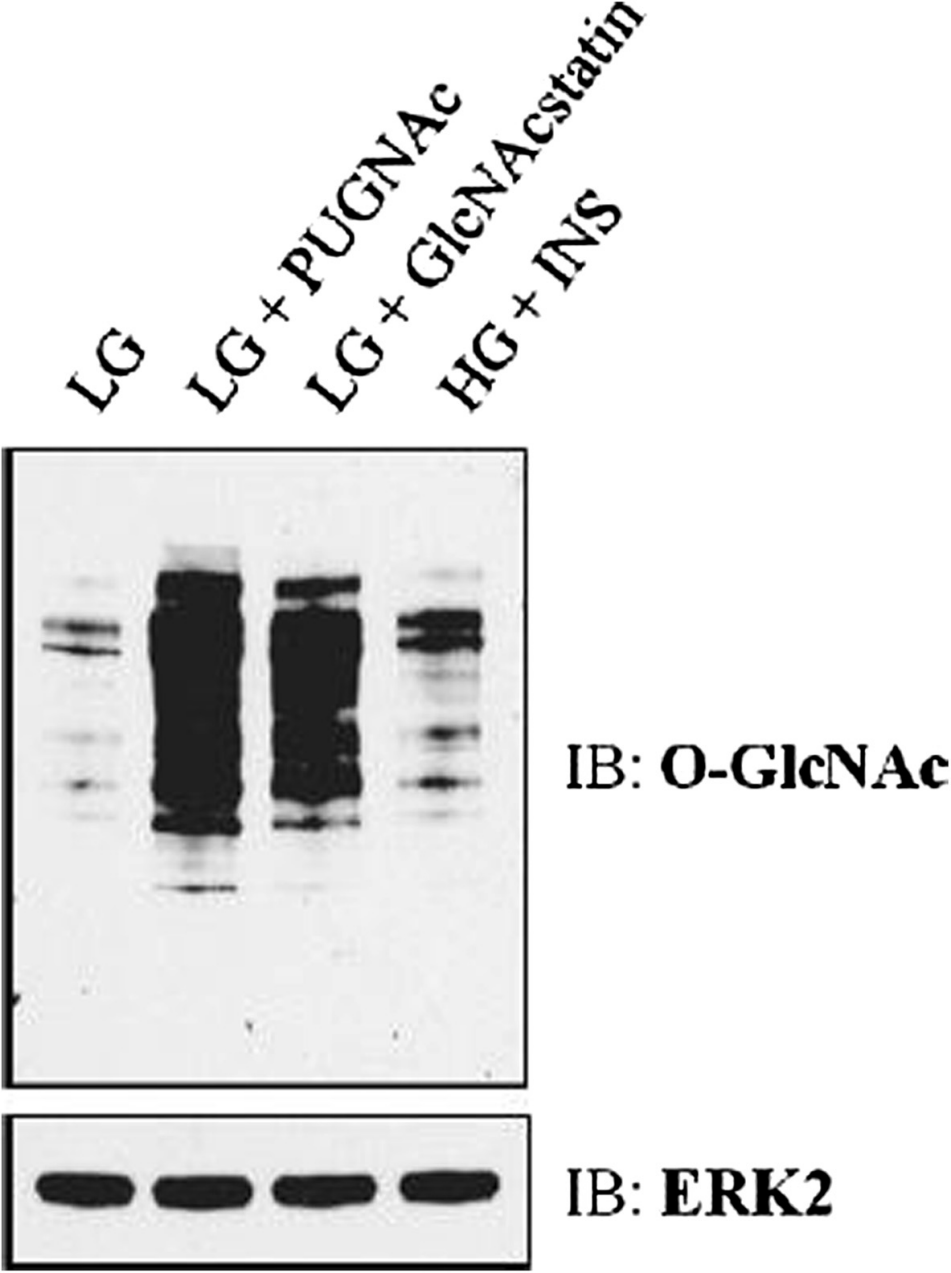
**Detection of O-GlcNAc levels in primary human adipocytes.** Global O-GlcNAc levels are elevated in three insulin resistant conditions generated by low glucose plus PUGNAc (LG + PUGNAc), low glucose plus GlcNAcstatin (LG + GlcNAcstatin), or high glucose plus chronic insulin exposure (HG + INS).

**Figure 2:**
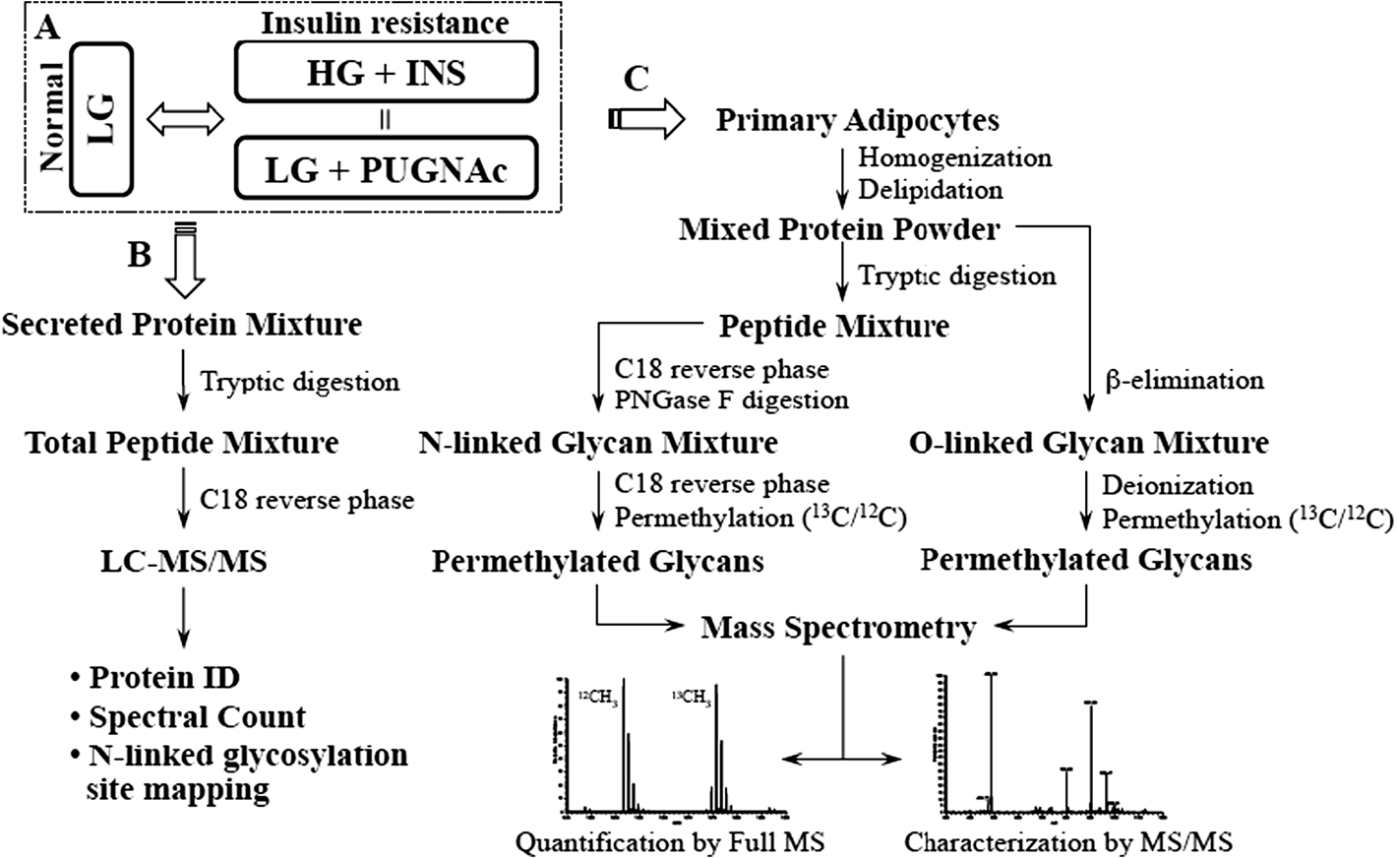
**Schematic flow diagram of the experimental procedure. (A)** Primary human adipocytes are treated with insulin responsive (LG, normoglycemic) or two insulin resistance generating conditions (HG + INS and LG + PUGNAc). **(B)** Identification and quantification of the secretory proteome and N-linked glycosylation site-mapping from the conditioned media of treated primary human adipocytes **(C)** Characterization and quantification of N- and O-linked glycans from a whole protein extract of treated primary human adipocytes.

Treatment with hyperglycemia and chronic insulin or normoglycemia and the pharmacological OGA inhibitor, PUGNAc, result in the global elevation of O ***-*** GlcNAc modified nucleocytosolic proteins and insulin resistance in rodent adipocytes [19,57]. We tested these conditions as well as treatment with a more specific OGA inhibitor, GlcNAcstatin, in primary human adipocytes [58]. Cryopreserved human preadipocytes were expanded and differentiated into mature adipocytes before treatment as described in *Materials and Methods*. Figure 1 shows that when compared to normoglycemia (LG), treatment with OGA inhibitors (LG + PUGNAc or LG + GlcNAcstatin) or treatment with hyperglycemia and hyperinsulinemia (HG + INS) raises global O-GlcNAc levels as measure by immunoblotting with an O-GlcNAc specific antibody. In a previous study using rodent adipocytes, we characterized and quantified the secretome and glycome during insulin sensitive and insulin resistant conditions induced by indirectly (HG + INS) or directly (LG + PUGNAc) modulating O-GlcNAc levels. We found that many secreted proteins were up or down-regulated upon the induction of insulin resistance. Here we took a parallel approach to ask the same question using primary human adipocytes. As shown in Figure 2, after treatment both the cells and conditioned media were harvested. Before the final 16 hour serum-free treatment incubation, the cells were washed five times with serum-free media to remove any traces of serum. This greatly reduced the complexity of the conditioned media. The conditioned media was buffer exchanged and concentrated before being processed for analysis by LC-MS/MS. Cells were collected and delipidated as in *Materials and Methods*. Protein was digested and prepared for either N-glycan or O-glycan analysis (Figure 2).

### Characterization of the primary human adipose tissue secretome by LC-MS/MS

**Figure 3:**
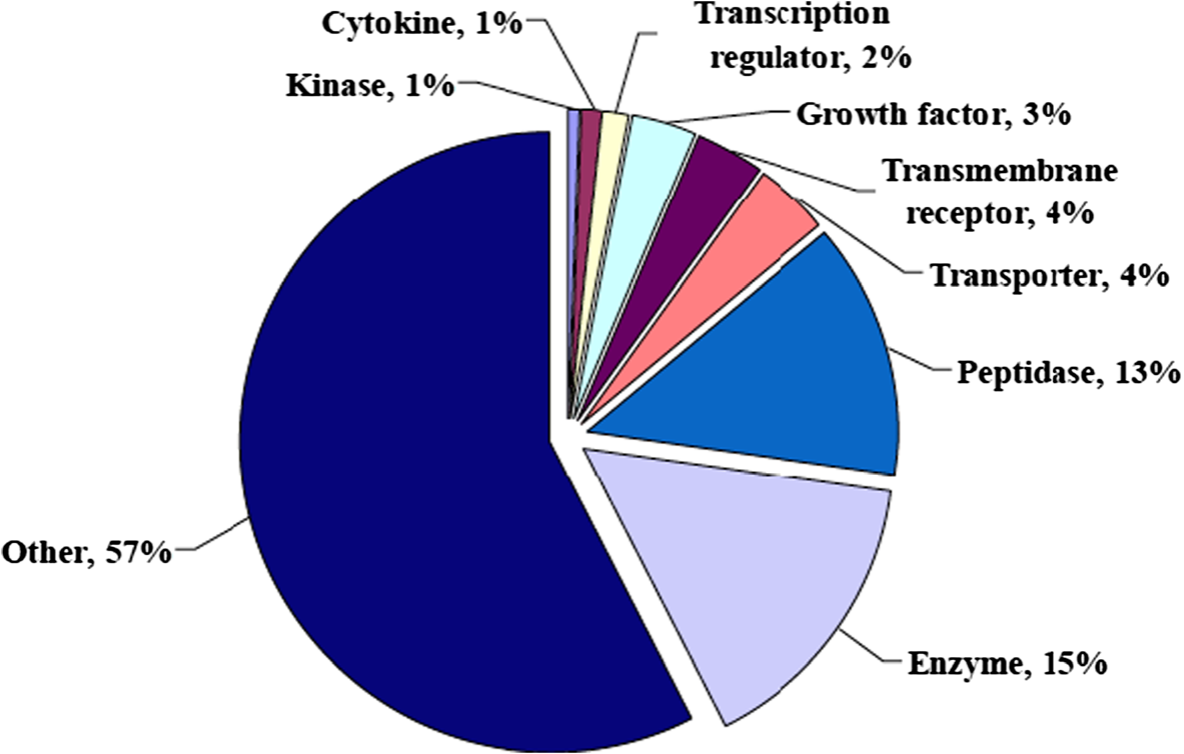
**The functional categories of the primary human adipocyte secretome.** The biological function analysis was determined for each protein based on the Ingenuity Pathway Analysis software (Ingenuity Systems).

The mass spectra from 24 mass spectrometric analyses were searched against a target nonredundant human database as well as a decoy human database using SEQUEST. Proteins identified by at least two non-redundant peptides with less than a 1% FDR were identified using ProteoIQ. Secreted proteins were manually determined by a reference search as described in *Materials and Methods*. Using these criteria, a total of 190 secreted proteins were identified. Table 1 shows the complete list of the 173 secreted proteins identified by at least 2 unique peptides. 17 of the secreted proteins identified based on a single peptide are presented in Supplemental Table 1 (Additional file 1: Table S1). Figure 3 shows the proportion of the secretome in each of the 9 main functional categories assigned by Ingenuity Pathways Analysis. The most highly represented categories are enzyme, peptidase, and other protein.

**Table 1:** Total secreted proteins from human dispose tissue by LC-MS/MS

**No.**	**Protein ID**	**Identifed proteins**	**Subcellular location**^**a**^
1	Q92484	Acid sphingomyelinase-like phosphodiesterase 3a	Extracellular
2	P07108	Acyl-CoA-binding protein	Cytoplasm
3	Q8IUX7	Adipocyte enhancer-binding protein 1	Membrane
4	P01023	Alpha-2-macroglobulin	Nucleus
5	P06733	Alpha-enolase	Extracellular
6	P15144	Aminopeptidase N	Cytoplasm
7	P01019	Angiotensinogen	Membrane
8	P07355	Annexin A2	Extracellular
9	P08758	Annexin A5	Membrane
10	Q8NCW5	Apolipoprotein A-I binding protein	Extracellular
11	P05090	Apolipoprotein D	Extracellular
12	P02649	Apolipoprotein E	Extracellular
13	P15289	Arylsulfatase A	Cytoplasm
14	P61769	Beta-2-microglobulin	Membrane
15	P21810	Biglycan	Extracellular
16	P43251	Biotinidase	Extracellular
17	P55290	Cadherin-13	Membrane
18	P27797	Calreticulin	Cytoplasm
19	O43852	Calumenin	Cytoplasm
20	P16870	Carboxypeptidase E	Membrane
21	P49747	Cartilage oligomeric matrix protein	Extracellular
22	P07858	Cathepsin B	Cytoplasm
23	P07339	Cathepsin D	Cytoplasm
24	P43235	Cathepsin K	Cytoplasm
25	P07711	Cathepsin L	Cytoplasm
26	Q9UBR2	Cathepsin Z	Cytoplasm
27	P36222	Chitinase-3-like protein 1	Extracellular
28	Q15782	Chitinase-3-like protein 2	Extracellular
29	Q59FG9	Chondroitin sulfate proteoglycan 2 (versican) variant	Extracellular
30	P10909	Clusterin	Extracellular
31	P02452	Collagen alpha-1(I) chain	Extracellular
32	P02458	Collagen alpha-1(II) chain	Extracellular
33	P02461	Collagen alpha-1(III) chain	Extracellular
34	P02462	Collagen alpha-1(IV) chain	Extracellular
35	P20908	Collagen alpha-1(V) chain	Extracellular
36	P12109	Collagen alpha-1(VI) chain	Extracellular
37	Q02388	Collagen alpha-1(VII) chain	Extracellular
38	P12107	Collagen alpha-1(XI) chain	Extracellular
39	Q99715	Collagen alpha-1(XII) chain	Extracellular
40	P39059	Collagen alpha-1(XV) chain [Contains: Endostatin]	Extracellular
41	P39060	Collagen alpha-1(XVIII) chain [Contains: Endostatin]	Extracellular
42	P08123	Collagen alpha-2(I) chain	Extracellular
43	P08572	Collagen alpha-2(IV) chain [Contains: Canstatin]	Extracellular
44	P05997	Collagen alpha-2(V) chain	Extracellular
45	P12110	Collagen alpha-2(VI) chain	Extracellular
46	P25940	Collagen alpha-3(V) chain	Extracellular
47	P12111	Collagen alpha-3(VI) chain	Extracellular
48	P08253	Collagenase (72 kDa type IV)	Extracellular
49	P00736	Complement C1r subcomponent	Extracellular
50	P09871	Complement C1s subcomponent	Extracellular
51	P01024	Complement C3	Extracellular
52	P29279	Connective tissue growth factor	Extracellular
53	Q9Y240	C-type lectin domain family 11 member A	Extracellular
54	O75462	Cytokine receptor-like factor 1	Extracellular
55	P07585	Decorin	Extracellular
56	Q07507	Dermatopontin	Extracellular
57	Q4VWZ6	Diazepam binding inhibitor, splice form 1c	Cytoplasm
58	Q9UBP4	Dickkopf-related protein 3	Extracellular
59	Q14118	Dystroglycan	Membrane
60	Q13822	Ectonucleotide pyrophosphatase/phosphodiesterase 2	Membrane
61	Q12805	EGF-containing fibulin-like extracellular matrix protein 1	Extracellular
62	O95967	EGF-containing fibulin-like extracellular matrix protein 2	Extracellular
63	Q9Y6C2	EMILIN-1	Extracellular
64	Q9BXX0	EMILIN-2	Extracellular
65	P61916	Epididymal secretory protein E1	Extracellular
66	Q9Y2E5	Epididymis-specific alpha-mannosidase	Cytoplasm
67	Q16610	Extracellular matrix protein 1	Extracellular
68	P08294	Extracellular superoxide dismutase [Cu-Zn]	Extracellular
69	P35555	Fibrillin-1	Extracellular
70	Q53TP5	Fibroblast activation protein, alpha subunit	Cytoplasm
71	Q06828	Fibromodulin	Extracellular
72	P02751	Fibronectin	Membrane
73	P23142	Fibulin-1	Extracellular
74	P98095	Fibulin-2	Extracellular
75	Q9UBX5	Fibulin-5	Extracellular
76	Q12841	Follistatin-related protein 1	Extracellular
77	P16930	Fumarylacetoacetase	Cytoplasm
78	P09382	Galectin-1	Extracellular
79	Q08380	Galectin-3-binding protein	Membrane
80	Q92820	Gamma-glutamyl hydrolase	Cytoplasm
81	P06396	Gelsolin	Extracellular
82	Q9UJJ9	GlcNAc-1-phosphotransferase subunit gamma	Cytoplasm
83	P07093	Glia-derived nexin	Extracellular
84	P04406	Glyceraldehyde-3-phosphate dehydrogenase	Cytoplasm
85	P35052	Glypican-1	Membrane
86	P28799	Granulins	Extracellular
87	Q14393	Growth-arrest-specific protein 6	Extracellular
88	P00738	Haptoglobin	Extracellular
89	P00739	Haptoglobin-related protein	Extracellular
90	O75629	Human Protein CREG1	Nucleus
91	P17936	Insulin-like growth factor-binding protein 3	Extracellular
92	P22692	Insulin-like growth factor-binding protein 4	Extracellular
93	P24592	Insulin-like growth factor-binding protein 6	Extracellular
94	Q16270	Insulin-like growth factor-binding protein 7	Extracellular
95	O95965	Integrin beta-like protein 1	Unknown
96	P19823	Inter-alpha-trypsin inhibitor heavy chain H2	Extracellular
97	O14498	ISLR	Extracellular
98	Q08431	Lactadherin	Extracellular
99	Q8NHP8	LAMA-like protein 2	Extracellular
100	Q59H37	Laminin alpha 2 subunit isoform b	Extracellular
101	Q16363	Laminin subunit alpha-4	Extracellular
102	P07942	Laminin subunit beta-1	Extracellular
103	P55268	Laminin subunit beta-2	Extracellular
104	P11047	Laminin subunit gamma-1	Extracellular
105	Q14767	Latent-transforming growth factor beta-binding protein 2	Extracellular
106	Q99538	Legumain	Cytoplasm
107	Q07954	Low-density lipoprotein receptor-related protein 1	Membrane
108	P51884	Lumican	Extracellular
109	P10619	Lysosomal protective protein	Cytoplasm
110	P13473	Lysosome-associated membrane glycoprotein 2	Membrane
111	Q9Y4K0	Lysyl oxidase homolog 2	Extracellular
112	P09603	Macrophage colony-stimulating factor 1	Extracellular
113	Q9UM22	Mammalian ependymin-related protein 1	Nucleus
114	P48740	Mannan-binding lectin serine protease 1	Extracellular
115	P50281	Matrix metalloproteinase-14	Extracellular
116	P01033	Metalloproteinase inhibitor 1	Extracellular
117	P16035	Metalloproteinase inhibitor 2	Extracellular
118	Q71SW6	Muscle type neuropilin 1	Membrane
119	P14543	Nidogen-1	Extracellular
120	Q14112	Nidogen-2	Extracellular
121	Q02818	Nucleobindin-1	Cytoplasm
122	Q9NRN5	Olfactomedin-like protein 3	Extracellular
123	Q86UD1	Out at first protein homolog	Unknown
124	P26022	Pentraxin-related protein PTX3	Extracellular
125	P62937	Peptidyl-prolyl cis-trans isomerase A	Cytoplasm
126	P23284	Peptidylprolyl isomerase B	Cytoplasm
127	Q15063	Periostin	Extracellular
128	P98160	Perlecan	Membrane
129	Q92626	Peroxidasin homolog	Unknown
130	P30086	Phosphatidylethanolamine-binding protein 1	Cytoplasm
131	P55058	Phospholipid transfer protein	Extracellular
132	P36955	Pigment epithelium-derived factor	Extracellular
133	Q9BTY2	Plasma alpha-L-fucosidase	Extracellular
134	Q9Y646	Plasma glutamate carboxypeptidase	Extracellular
135	P05155	Plasma protease C1 inhibitor	Extracellular
136	P05121	Plasminogen activator inhibitor 1	Extracellular
137	Q9GZP0	Platelet-derived growth factor D	Extracellular
138	P07602	Proactivator polypeptide [Contains: Saposin-A]	Extracellular
139	Q15113	Procollagen C-endopeptidase enhancer 1	Extracellular
140	Q02809	Procollagen-lysine,2-oxoglutarate 5-dioxygenase 1	Cytoplasm
141	P07737	Profilin-1	Cytoplasm
142	P41222	Prostaglandin-H2 D-isomerase	Cytoplasm
143	Q6UXB8	Protease inhibitor 16	Membrane
144	P07237	Protein disulfide-isomerase	Cytoplasm
145	Q15084	Protein disulfide-isomerase A6	Cytoplasm
146	P14618	Pyruvate kinase isozymes M1/M2	Cytoplasm
147	Q96D15	Reticulocalbin-3	Cytoplasm
148	Q99969	Retinoic acid receptor responder protein 2	Membrane
149	O75326	Semaphorin-7A	Membrane
150	Q12884	Seprase	Cytoplasm
151	Q92743	Serine protease HTRA1	Extracellular
152	P02787	Serotransferrin	Extracellular
153	P09486	SPARC	Extracellular
154	Q9BUD6	Spondin-2	Extracellular
155	Q9BRK5	Stromal cell-derived factor 4	Cytoplasm
156	O00391	Sulfhydryl oxidase 1	Cytoplasm
157	P00441	Superoxide dismutase [Cu-Zn]	Cytoplasm
158	Q9Y490	Talin-1	Membrane
159	P24821	Tenascin	Extracellular
160	P22105	Tenascin-X	Extracellular
161	Q08629	Testican-1	Extracellular
162	P10599	Thioredoxin	Cytoplasm
163	Q16881	Thioredoxin reductase 1	Cytoplasm
164	P07996	Thrombospondin-1	Extracellular
165	P35442	Thrombospondin-2	Extracellular
166	Q6FGX5	TIMP1 protein	Extracellular
167	Q15582	Transforming growth factor-beta-induced protein ig-h3	Extracellular
168	O14773	Tripeptidyl-peptidase 1	Cytoplasm
169	Q6EMK4	Vasorin	Membrane
170	P13611	Versican core protein	Extracellular
171	P08670	Vimentin	Cytoplasm
172	P04004	Vitronectin	Extracellular
173	O76076	WNT1-inducible-signaling pathway protein 2	Extracellular

^a^The subcellular location was determined for each protein based on the Ingenuity Pathway Analysis software (Ingenuity Systems).

### Quantification of the primary human adipocyte secretome

Several mass spectrometry techniques can be used for quantitative analysis. Both isotopic and non-isotopic labeling are commonly used to provide quantitative analysis of proteins in complex protein samples [59-67]. For non-isotopic or label-free MS-based quantitative analysis, ion chromatogram intensity or observed peptide spectral counts are compared using quantitative software tools [68-76]. Peptide spectral counts quantification relies on the association between protein abundance and the number of MS/MS spectra identifying each protein. For this study, we used three biological replicates of conditioned media samples, which differed in complex proteome profiles and relative number of normalized spectral counts. For each of the biological replicates, prepared conditioned media from three treatment groups (LG, LG + PUG, HG + INS) was subjected to two technical replicates of reverse phase LC-MS/MS. All together, a total of 18 LC-MS/MS experiments were performed. SEQUEST was used to search MS data files from each LC-MS/MS run against a forward and reversed human database to calculate a FDR. ProteoIQ was used to cluster the peptides to their assigned proteins and to gather normalized spectral counts for proteins identified at less than a 1% FDR and assigned by at least two non-redundant peptides. Spectral counts were normalized for each technical replicate. For each biological replicate the normalized spectral counts were averaged followed and then the comparative ratios between the two insulin resistant conditions and the insulin responsive condition were calculated. Finally, the comparative ratios were averaged between all of the biological replicates. We set the threshold for reporting statistically significant differences at ≥150% (1.5-fold), an average of ≥5 total spectral counts, and an average of ≥3 normalized spectral counts for each treatment group. We identified 20 human adipocytokines whose secretion levels were upregulated and 4 proteins that were downregulated by at least 1.5-fold by the transition from the insulin sensitive condition to both insulin resistant conditions (Table 2). We also identified 28 and 8 proteins that were upregulated or downregulated, respectively, in the transition from insulin sensitive to one of the insulin resistant conditions (Additional file 1: Table S2).

**Table 2:** **Human adipocytokines regulated a minimum of 150% under both insulin resistant conditaions**^**a**^

**No.**	**Protein ID**	**Identified proteins**	**LGPUG/LG**	**HGINS/LG**	**Ave. SC**	**SD**	**Ave. peptides**	**SD**
1	Q96D15	Reticulocalbin-3	9.00	8.52	11.50	2.89	3.00	1.15
2	Q9NRN5	Olfactomedin-like protein 3	5.40	7.57	13.60	6.31	3.00	0.00
3	P09603	Macrophage colony-stimulating factor 1	2.98	5.02	5.00	1.87	1.60	0.55
4	Q59H37	Laminin alpha 2 subunit isoform b	1.73	4.60	11.50	6.45	4.50	1.91
5	Q15113	Procollagen C-endopeptidase enhancer 1	1.53	4.11	14.00	3.56	3.75	0.50
6	Q07507	Dermatopontin	2.01	2.93	10.40	2.88	2.00	0.00
7	Q02809	Procollagen-lysine,2-oxoglutarate 5-dioxygenase 1	2.70	2.80	11.17	4.83	3.83	0.98
8	P36222	Chitinase-3-like protein 1	1.49	2.68	26.00	8.05	6.33	0.52
9	P05997	Collagen alpha-2(V) chain	1.55	2.44	9.33	2.89	2.67	0.58
10	Q9Y240	C-type lectin domain family 11 member A	2.07	2.44	11.00	2.00	3.00	0.00
11	Q06828	Fibromodulin	1.76	2.38	15.75	2.06	2.00	1.15
12	O00391	Sulfhydryl oxidase 1	2.20	2.27	8.17	3.06	2.67	0.52
13	P15144	Aminopeptidase N	2.19	2.03	22.00	13.34	4.33	1.86
14	Q99538	Legumain	1.60	1.92	11.67	4.18	1.83	0.98
15	Q6UXB8	Protease inhibitor 16	1.97	1.75	15.75	1.50	2.50	0.58
16	P09486	SPARC	1.48	1.74	263.00	169.53	10.33	1.37
17	P07942	Laminin subunit beta-1	1.48	1.69	63.67	13.34	16.83	1.72
18	O75326	Semaphorin-7A	1.95	1.66	21.17	8.89	3.67	1.03
19	P10909	Clusterin	1.77	1.63	17.00	6.39	3.17	0.75
20	P24821	Tenascin	1.61	1.54	37.33	4.76	9.83	1.72
**No.**	**Protein ID**	**Identified proteins**	**LG/LGPUG**	**LG/HGINS**	**Ave. SC**	**SD**	**Ave. peptides**	**SD**
1	P61769	Beta-2-microglobulin	2.40	1.90	68.17	35.76	3.17	0.98
2	P00739	Haptoglobin-related protein	1.87	1.66	5.67	0.58	2.00	0.00
3	Q16270	Insulin-like growth factor-binding protein 7	1.75	1.59	19.00	9.76	4.33	1.03
4	O95965	Integrin beta-like protein 1	1.68	1.57	9.00	1.41	3.00	0.00

^a^LGPUG: low glucose pluse PUGNAc; HGINS: high glucose pluse insulin as two insulin resistant conditions; Ave. SC: average total spectral count and SD: standard deviation.

**Figure 4:**
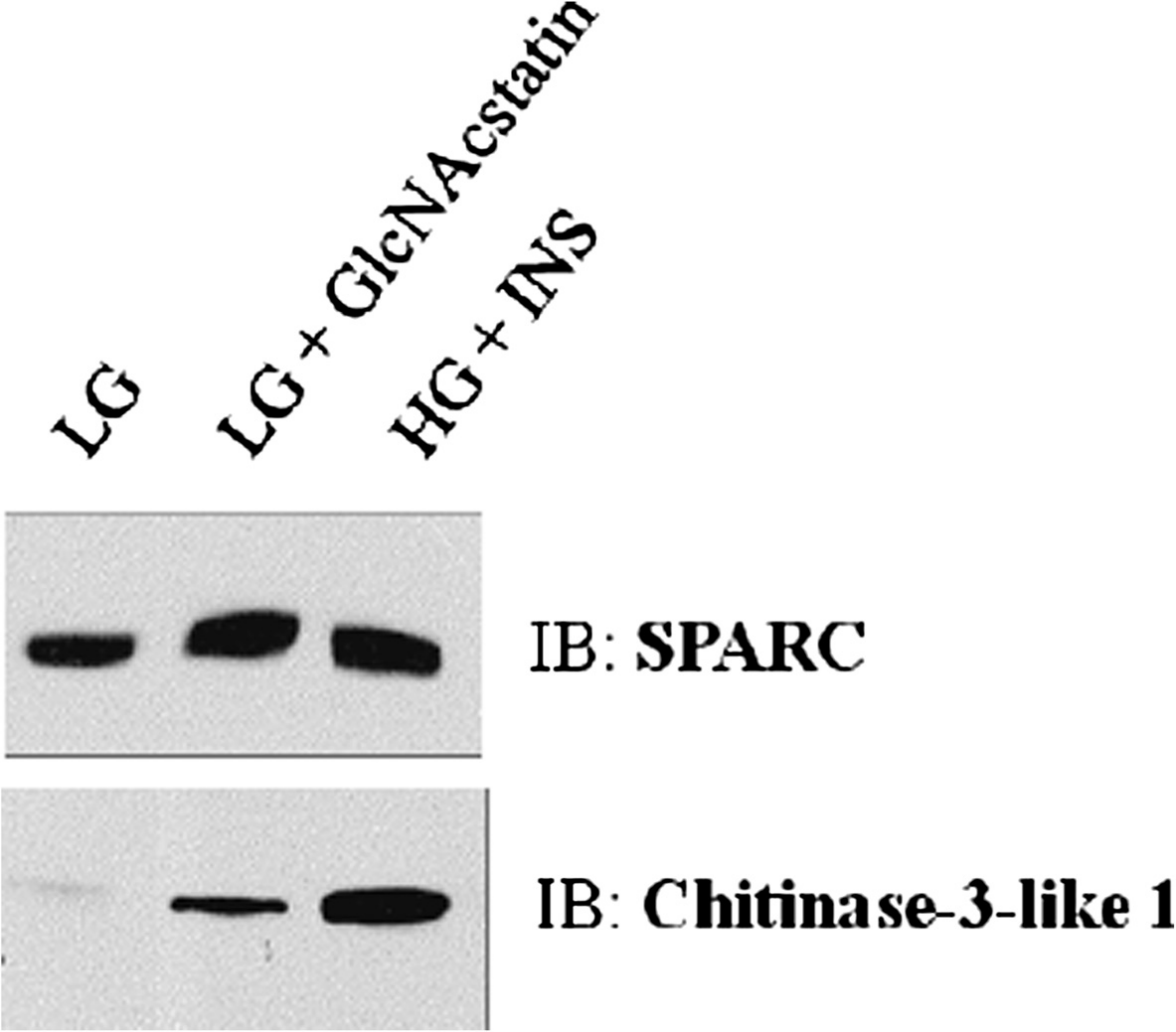
**Orthogonal validation of proteomic quantification.** Equal amounts of conditioned media from primary human adipocytes was immunoblotted with antibodies against SPARC or Chitinase-3-like protein 1.

To confirm the validity of the ratio that we used for statistical significance, we used the orthogonal method of immunoblotting. We confirmed that the regulation we observed in the proteomic experiment with immunoblotting for two proteins whose levels changed by only 1.5-fold in at least one of the insulin resistant conditions as determined by proteomics. In addition, we used cells where insulin resistance was induced using a more specific OGA inhibitor, GlcNAcstatin, to confirm the regulation we observed by using the less specific OGA inhibitor, PUGNAc [58,77]. Figure 4 shows that both SPARC and Chitinase-3-like protein 1 are upregulated in both insulin resistant conditions as determined by immunoblotting, which confirms our proteomic experiment.

### N-glycan site-mapping of the human adipocyte secretome

Many secreted proteins are modified by N-glycans. During disease states, the glycoforms of these proteins may change and have the potential to be used as disease biomarkers. Since the glycome of adipocytes is not well-defined, we determine sites of N-linked glycosylation on secreted adipocytokines as an initial step in biomarker discovery. The tryptic peptides generated from conditioned media were digested with PNGase F in the presence of ^18^O water to convert any N-glycan-modified Asp to an ^18^O-Asp residue. The resulting peptides were analyzed by LC-MS/MS using the previously described method for ITMS and the parent mass list method for FTMS as described in *Materials and Methods*. To obtain the parent mass list, the protein sequences of the 190 identified secreted proteins from Table 1 and Additional file 1: Table S1 were extracted from the database. From these sequences, tryptic peptides, with allowance for two internal missed cleavage sites, that contained the consensus sequence, N-X-S/T, were obtained for theoretical mass calculations. Table 3 shows a total of 91 N-linked glycosylation sites that were identified on 52 proteins. These sites covered 10.3% of the theoretical 882 N-linked glycosylation site sequences generated from the secreted protein list.

**Table 3:** **Identification of N-linked glycosylation sites using PNGase F with the incorporation of **^**18**^**O water in human adipocytokines**

**No.**	**Protein ID**	**Identifed proteins**	**N-linked peptides**^**a**^
1	P15144	Aminopeptidase N	KLN@YTLSQGHR
2	P21810	Biglycan	LLQVVYLHSNN@ITK
3	P16870	Carboxypeptidase E	GN@ETIVNLIHSTR
4	P07339	Cathepsin D	GSLSYLN@VTR
5	P43235	Cathepsin K	SN@DTLYIPEWEGR
6	P07711	Cathepsin L	YSVAN@DTGFVDIPK
7	P07711	Cathepsin L	YSVAN@DTGFVDIPKQEK
8	P10909	Clusterin	LAN@LTQGEDQYYLR
9	P02461	Collagen alpha-1(III) chain	ASQN@ITYHCK
10	P02461	Collagen alpha-1(III) chain	DGSPGGKGDRGEN@GSPGAPGAPGHPGPPGPVGPAGK
11	P20908	Collagen alpha-1(V) chain	VYCN@FTAGGSTCVFPDKK
12	P12109	Collagen alpha-1(VI) chain	ENYAELLEDAFLKN@VTAQICIDKK
13	P12109	Collagen alpha-1(VI) chain	GEDGPAGN@GTEGFPGFPGYPGNR
14	P12109	Collagen alpha-1(VI) chain	N@FTAADWGQSR
15	P12109	Collagen alpha-1(VI) chain	RN@FTAADWGQSR
16	Q02388	Collagen alpha-1(VII) chain	TAPEPVGRVSRLQILN@ASSDVLR
17	Q99715	Collagen alpha-1(XII) chain	EAGN@ITTDGYEILGK
18	P08123	Collagen alpha-2(I) chain	LLANYASQN@ITYHCK
19	P05997	Collagen alpha-2(V) chain	EASQN@ITYICK
20	P12111	Collagen alpha-3(VI) chain	GNPGEPGLN@GTTGPKGIR
21	P12111	Collagen alpha-3(VI) chain	GPPGVN@GTQGFQGCPGQR
22	P12111	Collagen alpha-3(VI) chain	GYPGDEGGPGERGPPGVN@GTQGFQGCPGQR
23	P09871	Complement C1s subcomponent	NCGVN@CSGDVFTALIGEIASPNYPKPYPENSR
24	O75462	Cytokine receptor-like factor 1	VLN@ASTLALALANLN@GSR
25	O75462	Cytokine receptor-like factor 1	VVDDVSN@QTSCR
26	P07585	Decorin	IADTN@ITSIPQGLPPSLTELHLDGNK
27	P07585	Decorin	LGLSFNSISAVDN@GSLANTPHLR
28	Q9UBP4	Dickkopf-related protein 3	GSN@GTICDNQR
29	Q13822	Ectonucleotide pyrophosphatase/phosphodiesterase 2	AEGWEEGPPTVLSDSPWTN@ISGSCK
30	Q13822	Ectonucleotide pyrophosphatase/phosphodiesterase 2	AIIAN@LTCK
31	Q9Y6C2	EMILIN-1	LGALN@SSLQLLEDR
32	P35555	Fibrillin-1	TAIFAFN@ISHVSNK
33	P02751	Fibronectin	DQCIVDDITYNVN@DTFHKR
34	P02751	Fibronectin	DQCIVDDITYNVN@DTFHK
35	P02751	Fibronectin	LDAPTNLQFVN@ETDSTVLVR
36	Q12841	Follistatin-related protein 1	GSN@YSEILDK
37	Q12841	Follistatin-related protein 1	GSN@YSEILDKYFK
38	P09382	Galectin-1	FNAHGDANTIVCNSK
39	Q08380	Galectin-3-binding protein	ALGFEN@ATQALGR
40	Q08380	Galectin-3-binding protein	DAGVVCTN@ETR
41	Q08380	Galectin-3-binding protein	TVIRPFYLTN@SSGVD
42	P00738	Haptoglobin	VVLHPN@YSQVDIGLIK
43	O75629	Human Protein CREG1	LN@ITNIWVLDYFGGPK
44	P17936	Insulin-like growth factor-binding protein 3	GLCVN@ASAVSR
45	O14498	ISLR	SLDLSHNLISDFAWSDLHN@LSALQLLK
46	Q8NHP8	LAMA-like protein 2	SDLNPAN@GSYPFKALR
47	Q16363	Laminin subunit alpha-4	DAVRN@LTEVVPQLLDQLR
48	Q16363	Laminin subunit alpha-4	FYFGGSPISAQYAN@FTGCISNAYFTR
49	Q16363	Laminin subunit alpha-4	LITEEAN@R
50	Q16363	Laminin subunit alpha-4	LTLSELDDIIKN@ASGIYAEIDGAK
51	Q16363	Laminin subunit alpha-4	RPASN@VSASIQR
52	P07942	Laminin subunit beta-1	LSDTTSQSN@STAK
53	P11047	Laminin subunit gamma-1	VN@NTLSSQISR
54	P11047	Laminin subunit gamma-1	KYEQAKN@ISQDLEK
55	P11047	Laminin subunit gamma-1	LLNN@LTSIK
56	P11047	Laminin subunit gamma-1	TAN@DTSTEAYNLLLR
57	P11047	Laminin subunit gamma-1	TLAGEN@QTAFEIEELNR
58	P11047	Laminin subunit gamma-1	VNDN@KTAAEEALR
59	Q14767	Latent-transforming growth factor beta-binding protein 2	DGTQQAVPLEHPSSPWGLN@LTEK
60	P51884	Lumican	AFEN@VTDLQWLILDHNLLENSK
61	P51884	Lumican	LGSFEGLVN@LTFIHLQHNR
62	P51884	Lumican	LHINHNN@LTESVGPLPK
63	P13473	Lysosome-associated membrane glycoprotein 2	IAVQFGPGFSWIAN@FTK
64	P01033	Metalloproteinase inhibitor 1	AKFVGTPEVN@QTTLYQR
65	P01033	Metalloproteinase inhibitor 1	FVGTPEVN@QTTLYQR
66	P01033	Metalloproteinase inhibitor 1	SHN@RSEEFLIAGK
67	Q9NRN5	Olfactomedin-like protein 3	IYVLDGTQN@DTAFVFPR
68	P26022	Pentraxin-related protein PTX3	ATDVLN@K
69	Q15063	Periostin	EVN@DTLLVNELK
70	Q15063	Periostin	IFLKEVN@DTLLVNELK
71	P98160	Perlecan	SLTQGSLIVGDLAPVN@GTSQGK
72	P55058	Phospholipid transfer protein	VSN@VSCQASVSR
73	P36955	Pigment epithelium-derived factor	VTQN@LTLIEESLTSEFIHDIDR
74	P36955	Pigment epithelium-derived factor	VTQN@LTLIEESLTSEFIHDIDRELK
75	P05155	Plasma protease C1 inhibitor	VGQLQLSHN@LSLVILVPQNLK
76	P05155	Plasma protease C1 inhibitor	VLSN@NSDANLELINTWVAK
77	P07602	Proactivator polypeptide [Contains: Saposin-A]	TN@STFVQALVEHVK
78	P07602	Proactivator polypeptide [Contains: Saposin-A]	LIDNN@KTEK
79	P07602	Proactivator polypeptide [Contains: Saposin-A]	LIDNN@KTEKEILDAFDK
80	P07602	Proactivator polypeptide [Contains: Saposin-A]	NLEKN@STK
81	P07602	Proactivator polypeptide [Contains: Saposin-A]	NLEKN@STKQEILAALEK
82	P07602	Proactivator polypeptide [Contains: Saposin-A]	TN@STFVQALVEHVKEECDR
83	P09486	SPARC	VCSNDN@K
84	P09486	SPARC	VCSNDN@KTFDSSCHFFATK
85	P24821	Tenascin	N@TTSYVLR
86	P24821	Tenascin	LN@YSLPTGQWVGVQLPR
87	P07996	Thrombospondin-1	VVN@STTGPGEHLR
88	P35442	Thrombospondin-2	VVN@STTGTGEHLR
89	Q6FGX5	TIMP1 protein	FVGTPEVN@QTTLYQR
90	Q6FGX5	TIMP1 protein	SHN@RSEEFLIAGK
91	Q6EMK4	Vasorin	LHEITN@ETFR

^a^An @ indicates the site of N-linked glycosylation.

### Characterization of the human adipocyte glycome

**Figure 5:**
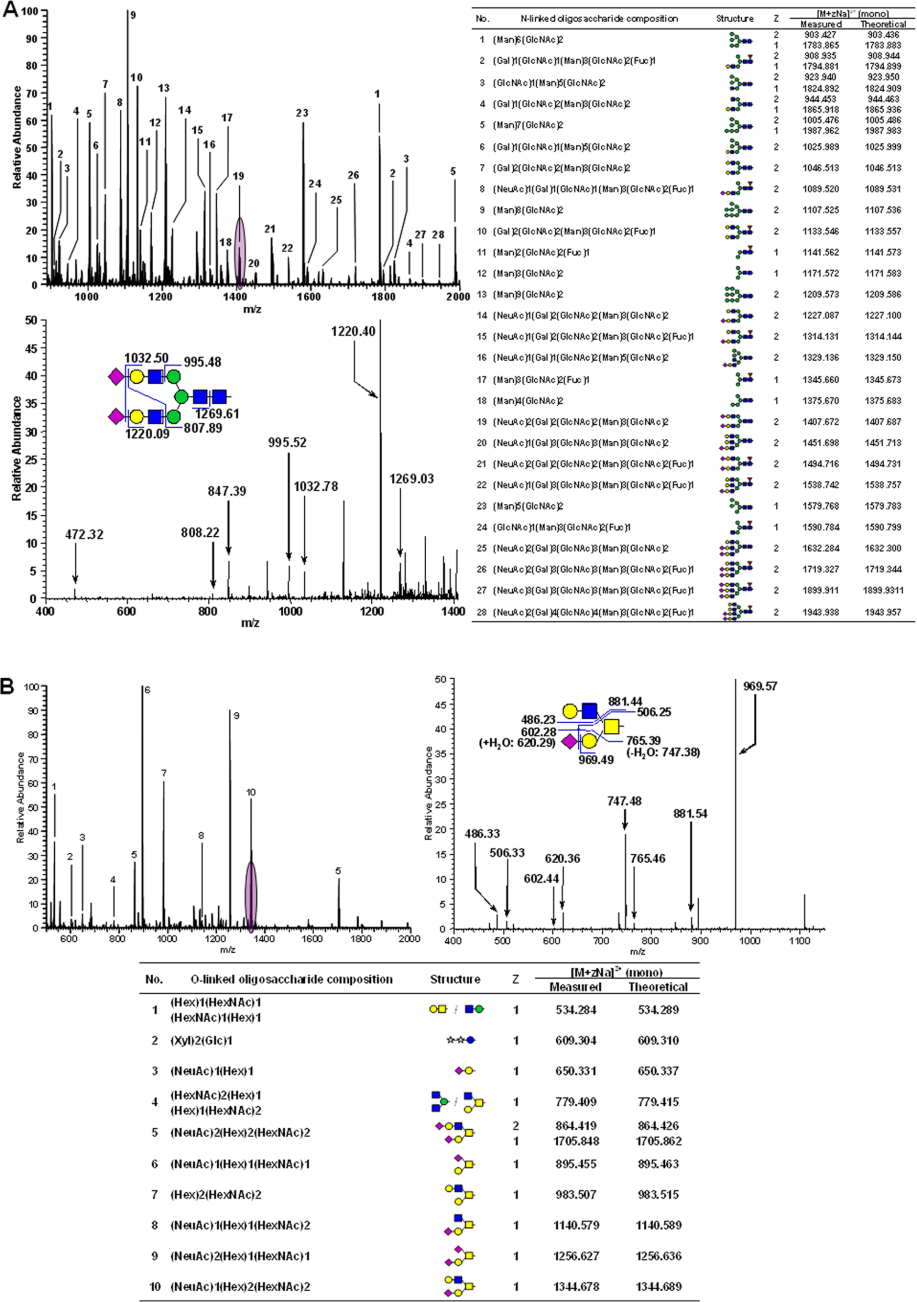
**Characterization of the primary human adipocyte glycome by MS/MS and TIM scan. (A)** Upper left panel: a full FTMS spectrum of the N-linked glycan mixture, lower left panel: the characterization of a biantennary complex N-linked glycan structure by MS/MS fragmentation, right panel: a list of predominant N-linked glycans. **(B)** Upper left panel: a full FTMS spectrum of the O-linked glycan mixture, upper right panel: the characterization of a core 2 O-linked glycan structure by MS/MS fragmentation, lower panel: a list of predominant O-linked glycans. (pink star): Xyl, (red inverted triangle): Fuc, (blue circle): Glc, (green circle): Man, (yellow circle): Gal, (blue square): GlcNAc, (yellow square): GalNAc, and (violet diamond): NeuAc.

To better define the glycome of human adipocytes, we performed N-linked and O-linked glycan analysis using mass spectrometry. Glycans were released from whole-cell human adipocyte extracts by PNGase F for N-linked glycans and by β-elimination for O-linked glycans as shown in Figure 2C. Figure 5A shows that the permethylated N-linked glycans were characterized by full FTMS spectrum using a LTQ Orbitrap XL *(left panel, top)* followed by MS/MS fragmentation by TIM analysis in the ion trap *(left panel, bottom)*. The fragmentation of a biantennary N-linked glycan is shown as an example. We used the GlycoWorkbench to manually interpret a total of 155 N-linked glycan structures from MS/MS spectra at different charge states as shown in Additional file 1: Table S3. In Figure 5A *(right panel)*, we show that 28 of the predominant N-linked glycans are assigned to a full FTMS and a MS/MS spectrum.

Figure 5B *(left panel)* shows a full FTMS spectrum from an O-linked glycan mixture. 10 of the predominant O-linked glycans were assigned from the full FTMS spectra (Figure 5B, *bottom panel*). A representative fragmentation spectrum is shown for one of the core 2 O-linked glycans (Figure 5B, *right panel*). A total of 29 O-linked glycans were characterized from MS/MS spectra at singly and doubly charged states as shown in Additional file 1: Table S4.

### Relative quantification of the human adipocyte glycome upon the induction of insulin resistance using ^13^C labeling and prevalence

**Figure 6:**
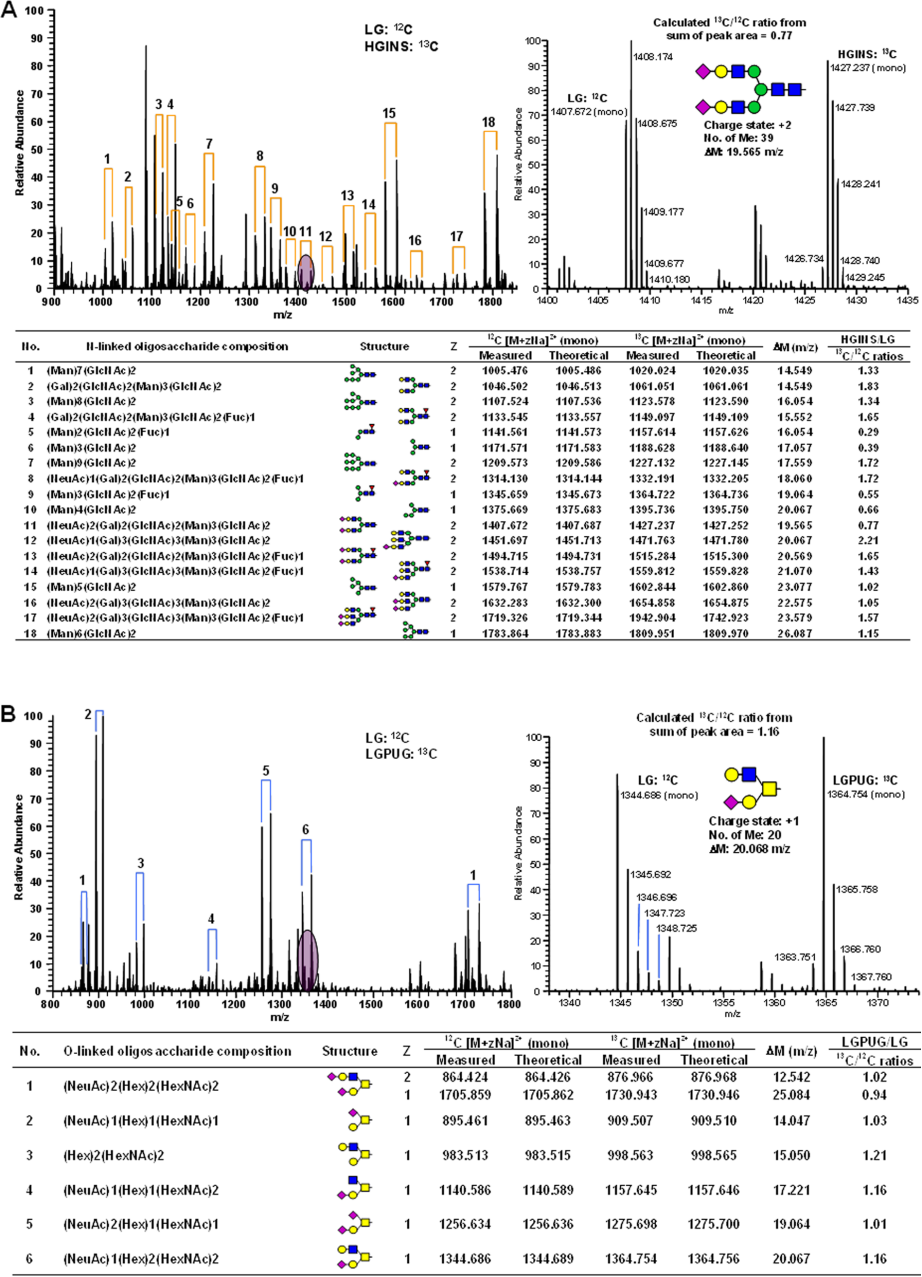
**Relative quantification of the primary human adipocytes glycome during insulin resistance using **^**13**^**C/**^**12**^**C labeling. (A)** Upper left panel: a full FTMS spectrum of ^13^C/^12^C labeled N-linked glycans in HGINS and LG, upper right panel: a FTMS spectrum to calculate ^13^C/^12^C ratios from the sum of isotopic peak areas between the isotopic pairs, lower panel: a list of relative ratios for HGINS and LG for predominant N-linked glycans. **(B)** Upper left panel: a full FTMS spectrum of ^13^C/^12^C labeled O-linked glycans in LGPUG and LG, upper right panel: a FTMS spectrum to calculate ^13^C/^12^C ratios from the sum of isotopic peak areas between the isotopic pairs, lower panel: a list of relative ratios for LGPUG and LG for predominant O-linked glycans.

For the comparative quantification of glycans, we performed isotopic labeling using heavy/light iodomethane (^13^CH_3_I and ^12^CH_3_I) [78,79]. The ^13^C/^12^C ratio from the sum of the peak areas and the prevalence ratio between the different treatment conditions were used for relative quantification. Permethylated glycans were mixed in a 1:1 protein ratio and analyzed in quadruplicate using an LTQ Orbitrap XL. Figure 6A (*left panel*) shows the isotopic pairs of N-linked glycans on a full FTMS spectrum. Figure 6A (*right panel*) shows an example of a calculated ^13^C/^12^C ratio from sum of peak area between LG + PUGNAc (LGPUG) and LG conditions. Additional file 1: Table S5 shows a total of 48 N-linked glycans that were relatively quantified between the insulin resistant conditions (LGPUG and HGINS) and insulin responsive condition (LG) by the average ^13^C/^12^C ratio and the average prevalence ratio. This approach simultaneously quantified a broad range of N-glycan structures in a complex mixture; however we did not observe highly significant changes in N-glycan ^13^C/^12^C ratios or prevalence ratios. Figure 6B (*left panel*) shows the isotopic pairs of O-linked glycans on a full FTMS spectrum. Figure 6B (*right panel*) shows an example of the calculated ^13^C/^12^C ratio from the sum of peak areas between LGPUG and LG conditions. Additional file 1: Table S6 shows a total of 12 O-linked glycans that were relatively quantified between insulin resistant and insulin sensitive conditions. We did not observe highly significant changes in O-glycan ^13^C/^12^C ratios or prevalence ratios.

## Discussion

Several groups have reported the rodent adipocyte secretome during insulin resistance, but there have been fewer such studies performed with human adipocytes [25,80-82]. It is advantageous to investigate the secretome of human adipose tissue because there are bound to be differences between the rodent and human secretome [8]. The differences in the secretome of human adipose tissue have been investigated during adipogenesis and between different fat pad depots [83-86]. Other studies have characterized the secretome without quantification [87,88]. Another approach has been to study a limited list of known adipocytokines during insulin sensitivity and insulin resistance [89,90]. Therefore, our study fills an important gap in the literature by taking a discovery approach to characterizing and quantifying the human adipocyte secretome in both insulin sensitive and two different insulin resistance conditions. In addition, we characterize and quantify the glycome of human adipocytes, which has not been described to our knowledge.

We report a total of 193 secreted proteins from human adipocytes (Table 1 and Additional file 1: Table S1). Using Ingenuity Pathways Analysis we determined that many of the secreted proteins were enzymes, peptidases, or other functions (Figure 3). The largest pool of identified proteins from the other category corresponds to extracellular matrix proteins. A common theme among the adipose tissue secretome studies is that extracellular matrix (ECM) proteins, ECM remodelers, inflammatory, and angiogenesis proteins are highly represented [7,81,87,91]. The accumulation of fat mass during obesity requires extensive tissue remodeling that is accomplished by ECM remodelers. The remodeling can generate an inflammatory response during obesity and insulin resistance due to limited angiogenesis, localized hypoxia, adipocyte necrosis, increased fibrosis, altered adipocytokine secretion, and M1-stage macrophage and other immune cell infiltration [4,92-96]. Many of the adipocytokines we identified, such as SPARC and Chitinase-3-like protein 1, have been implicated in inflammation [97,98]. In addition, tissue remodeling requires extensive crosstalk between the different cell types that comprise adipose tissue. Several of the adipocytokines we identified, such as Gelsolin, Calreticulin, and Cathepsin D, may be expressed in cell types other than adipocytes [87,99]. By using primary adipocytes instead of a cell line, the secretome we identified better represents the physiological status of adipose tissue. In the future, these identified adipocytokines may potentially be used as prognostic/diagnostic biomarkers for metabolic syndrome, T2DM, and other complications.

We generated insulin resistance in the human adipocytes by both directly and indirectly modulating O-GlcNAc levels. The elevation of O-GlcNAc levels has been shown to be sufficient to induce insulin resistance in many systems [19-24]. We have previously demonstrated that the induction of insulin resistance in this way in rodent adipocytes alters the secretion of several adipocytokines [25]. Here, we showed that 20 and 4 adipocytokines were upregulated or downregulated, respectively, upon the transition to insulin resistance by both directly and indirectly modulating O-GlcNAc levels (Table 2). We also identified 28 and 8 adipocytokines that were upregulated or downregulated, respectively, upon the induction of insulin resistance by one of the two methods (Additional file 1: Table S2). In this study, we find that Sulfhydryl oxidase 1 (Quiescin Q6), laminin B1, and Chitinase-3-like protein 1 are upregulated during both insulin resistant conditions, which was also observed in the rodent study. SPARC was upregulated in both insulin resistant conditions in human adipocytes and was found to be upregulated in HG + INS in rodent adipocytes by proteomics. Four other proteins were observed to be regulated by insulin resistance in both rodent and human adipocytes; however, the regulation was slightly different between species. Thioredoxin was downregulated in both insulin resistant conditions in rodent adipocytes and during HG + INS in human adipocytes but was upregulated during LG + PUG in human adipocytes. Gelsolin was upregulated in both insulin resistant conditions in rodent adipocytes and during HG + INS in human adipocytes but was not regulated by LG + PUG. Fibulin 2 was downregulated in both insulin resistant conditions in rodent adipocytes but was not regulated or upregulated in LG + PUG and HG + INS, respectively, in human adipocytes. From these examples, it is clear that there are differences in adipocytokine secretion between rodents and humans. Because the proteome sample is extremely complex, we may not have achieved sufficient resolution to quantify some of the proteins that were observed in both rodent and human adipocyte secretomes, such as Angiotensinogen, Cathepsin B, and Spondin 1 [25,100]. Because we assigned significance to a relatively small fold change (1.5 fold) for quantification, we verified the regulation of two adipocytokines that changed by 1.5 fold by an independent, orthogonal method. Both SPARC and Chitinase-3-like protein 1 protein levels were altered in a similar manner as measured by both quantitative proteomics and immunoblotting (Figure 4). In addition, the regulation of these proteins by O-GlcNAc was verified using a more OGA-specific N-acetyglucosaminidase inhibitor, GlcNAcstatin.

The inclusion of an insulin resistance condition generated by directly modulating O-GlcNAc levels in our proteomic study helped us to better define the proteins regulated by O-GlcNAc in human adipocytes. The O-GlcNAc modification is found on hundreds in nucleocytosolic proteins and can affect protein function in diverse ways, such as protein-protein interactions, protein degradation, interplay with phosphorylation, localization, and transcriptional activation [101]. Compiling a list of proteins modulated by O-GlcNAc is the first step in understanding the mechanism of how O-GlcNAc affects adipocytokine expression. We have used the data generated by our previous rodent proteomic study to investigate the transcriptional regulation of adipocytokines by global O-GlcNAc levels (unpublished data). Although several studies have associated elevated O-GlcNAc levels with adipocytokine expression, no specific molecular mechanism has been reported [13-16,21,102,103].

We also characterize the N-linked and O-linked glycome for human adipocytes. Glycosylation and other PTM’s provide an additional layer of regulatory complexity to expressed proteins. To understand the full functionality of a protein, one must know the state of the PTM’s. Since secretory proteins, such as adipocytokines, are exposed to the glycosylation machinery that reside in the ER and Golgi apparatus as they make their way through the secretory pathway, they often have a high degree of complex glycosylation. The subset of glycans expressed by cells depends on multiple factors, such as developmental stage, tissue type, and the genetic and physiological state of the cell. It is not known whether adipocytes change glycan expression patterns in response to insulin resistance. Since glycosylation can affect diverse processes that are relevant to adipose tissue remodeling in obesity, such as cell-cell interactions, growth factor sequestration, cytokine activation, and cell migration, there is a need to define the adipocyte glycome [34,104,105].

Characterizing the glycome poses many challenges because there is no template for modification, as in DNA, and there is an incredible diversity of glycoforms. Mass spectrometry can provide both structural and abundance information using a small amount of material from a relatively complex mixture [106]. However, it is still difficult to assign specific glycoforms to specific protein residues in a complex mixture. To determine sites of N-glycosylation on the secreted proteins, we used PNGase F and ^18^O water to convert the glycan-modified Asn to an ^18^O-Asp residue, thereby leaving a chemical marker of the glycosylation site. We identified 91 sites on 51 proteins of N-linked glycosylation by Orbitrap mass spectrometer using the parent mass list obtained from theoretical consensus sequence in the secretome list (Table 3). For relative glycan quantification, we permethylated using heavy or light iodomethane (^13^CH_3_I and ^12^CH_3_I) and used prevalence data. The use of isotopic labels allows for mixing of the samples to be compared before analysis, thus providing an internal standard and preventing run to run technical variation [49,78]. These techniques allow us to determine the full diversity and changes in major and minor adipocyte glycans during insulin sensitive and resistant conditions. We characterized a total of 155 N-linked glycans and 29 of O-linked glycans (Figure 5, Additional file 1: Table S3, Table S4). The results presented here demonstrate that the human adipocytes are expressing extended hybrid and complex N-linked glycans, in addition to high mannose glycans. Adipose tissues possess the biosynthetic ability to generate diverse O-linked glycans such as core structure, O-glucose glycans, and O-fucose glycans.

Following characterization, we determined the change in glycan prevalence during insulin resistance using isotopic and prevalence ratios. We quantified 48 N-linked glycans and 12 O-linked glycans (Figure 6, Additional file 1: Tables S5-S6). Our results showed that changes of glycan prevalence were not statistically different between the insulin sensitive and insulin resistant conditions. It has been suggested that altering the intracellular UDP-GlcNAc pool, such as during increased glucose flux, changes the degree of N-glycan branching in an ultrasensitive manner [37]. While it is clear that the induction of insulin resistance in adipocytes massively upregulates intracellular glycosylation (Figure 1), we did not observe significant changes in complex glycosylation. This suggests that changes in complex glycosylation are not necessary for the induction of insulin resistance; although we cannot rule out that they play a role in insulin resistance that occurs over a long period of time. Since insulin resistance was generated by treatment for 40 hours, it is possible that the changes in glycan structure had not had time to significantly change.

In conclusion, we have characterized the secretome and glycome of primary human adipocytes during insulin resistance using a proteomic approach. We generated insulin resistance by either directly or indirectly modulating O-GlcNAc levels, which provides a list of adipocytokines that are modulated by O-GlcNAc levels. Adipocytokine and glycan levels were quantified between insulin sensitive and insulin resistant conditions and sites of N-glycosylation were identified. This study helps to define the secretome of primary human adipocytes during insulin resistance and helps to characterize and quantify the previously unknown primary adipocyte glycome. Finally, while elevated glucose levels and flux through the hexosamine pathway could theoretically alter both complex glycosylation and O-GlcNAc levels, our findings clearly demonstrate, under the short time frame used here where significant changes were observed in adipocytokine secretion but not in complex glycosylation, that only elevation in O-GlcNAc levels correlate with the observed changes in adipocytokine secretion.

## Competing interests

The authors declare that they have no competing interests.

## Authors’ contributions

JML participated in the design and execution of experiments and in the writing of the manuscript. EEW–H participated in the execution of experiments as in the writing of the manuscript. CFT and DH participated in the execution of experiments and editing of the manuscript. LW participated in the design of experiments and the writing and final editing of the manuscript. All authors read and approved the final manuscript.

## Supplementary Material

Additional file 1: Table S1. The list of secreted proteins identified by single peptide detection from primary human adipocytes. **Table S2.** Human adipocytokines regulated a minimum of 150% under one of the insulin resistant conditions. **Table S3.** Characterization of total N-linked glycans from primary human adipocytes by MS/MS and TIM scan. **Table S4.** Characterization of total O-linked glycans from primary human adipocytes by MS/MS and TIM scan. **Table S5.** Relative quantification of N-linked glycans from primary human adipocytes between insulin resistant conditions and insulin responsive condition by ^13^C/^12^C ratio and prevalence ratio. **Table S6.** Relative quantification of O-linked glycans from human adipocytes between insulin resistant conditions and insulin responsive condition by ^13^C/^12^C ratio and prevalence ratio. Click here for file
